# Antiviral Effect of Microalgae *Phaeodactylum tricornutum* Protein Hydrolysates against Dengue Virus Serotype 2

**DOI:** 10.3390/md22080369

**Published:** 2024-08-14

**Authors:** Bianca Vianey Rivera-Serrano, Sandy Lucero Cabanillas-Salcido, Carlos Daniel Cordero-Rivera, Ricardo Jiménez-Camacho, Claudia Desiree Norzagaray-Valenzuela, Loranda Calderón-Zamora, Luis Adrián De Jesús-González, José Manuel Reyes-Ruiz, Carlos Noe Farfan-Morales, Alejandra Romero-Utrilla, Víctor Manuel Ruíz-Ruelas, Josué Camberos-Barraza, Alejandro Camacho-Zamora, Alberto Kousuke De la Herrán-Arita, Carla Angulo-Rojo, Alma Marlene Guadrón-Llanos, Ángel Radamés Rábago-Monzón, Janitzio Xiomara Korina Perales-Sánchez, Marco Antonio Valdez-Flores, Rosa María Del Ángel, Juan Fidel Osuna-Ramos

**Affiliations:** 1 Faculty of Medicine, Autonomous University of Sinaloa, Culiacán 80246, Mexico; vianeyrivera.fm@uas.edu.mx (B.V.R.-S.); cabanillassandy.fm@uas.edu.mx (S.L.C.-S.); victor_199705@hotmail.com (V.M.R.-R.); josue.camberos@uas.edu.mx (J.C.-B.); alejandrocamacho@uas.edu.mx (A.C.-Z.); alberto.kousuke@uas.edu.mx (A.K.D.l.H.-A.); carla.angulo@uas.edu.mx (C.A.-R.); almaguadron@uas.edu.mx (A.M.G.-L.); rabagoradames.fm@uas.edu.mx (Á.R.R.-M.); 2 Programa de Maestría en Ciencias en Biomedicina Molecular, Facultad de Medicina, Universidad Autónoma de Sinaloa (UAS), Culiacán 80246, Mexico; 3 Department of Infectomics and Molecular Pathogenesis, Center for Research and Advanced Studies (CINVESTAV-IPN), Mexico City 07360, Mexico; carlos.cordero@cinvestav.mx (C.D.C.-R.); ricardo.jimenez@cinvestav.mx (R.J.-C.); 4 Faculty of Biology, Autonomous University of Sinaloa, Culiacán 80019, Mexico; claudia.norzagaray@uas.edu.mx (C.D.N.-V.); loranda.calderon@uas.edu.mx (L.C.-Z.); 5 Unidad de Investigación Biomédica de Zacatecas, Instituto Mexicano del Seguro Social (IMSS), Zacatecas 98000, Mexico; luis.dejesus@cinvestav.mx; 6 Unidad Médica de Alta Especialidad, Hospital de Especialidades No. 14, Centro Médico Nacional “Adolfo Ruiz Cortines”, Instituto Mexicano del Seguro Social (IMSS), Veracruz 91897, Mexico; jose.reyesr@imss.gob.mx; 7 Facultad de Medicina, Región Veracruz, Universidad Veracruzana, Veracruz 91700, Mexico; 8 Departamento de Ciencias Naturales, Universidad Autónoma Metropolitana (UAM), Unidad Cuajimalpa, Ciudad de México 05348, Mexico; cfarfan@cua.uam.mx; 9 Departamento de Anatomía Patológica, Instituto Mexicano del Seguro Social (IMSS), Culiacán 80200, Mexico; alejandraromeroutrilla@gmail.com; 10 Programa de Maestría en Ciencias en Medicina Traslacional y Salud Publica, Facultad de Medicina, Universidad Autónoma de Sinaloa (UAS), Culiacán 80246, Mexico; 11 Programa de Doctorado en Ciencias en Biomedicina Molecular, Facultad de Medicina, Universidad Autónoma de Sinaloa (UAS), Culiacán 80246, Mexico; 12 Bioprocesses Laboratory, Faculty of Chemical-Biological Sciences, Autonomous University of Sinaloa, Culiacán 80246, Mexico; janitzio.perales@uas.edu.mx

**Keywords:** DENV, peptides, antiviral, microalgae, *Phaeodactylum tricornutum*

## Abstract

Dengue, caused by the dengue virus (DENV), is a global health threat transmitted by *Aedes* mosquitoes, resulting in 400 million cases annually. The disease ranges from mild to severe, with potential progression to hemorrhagic dengue. Current research is focused on natural antivirals due to challenges in vector control. This study evaluates the antiviral potential of peptides derived from the microalgae *Phaeodactylum tricornutum*, known for its bioactive compounds. Microalgae were cultivated under controlled conditions, followed by protein extraction and hydrolysis to produce four peptide fractions. These fractions were assessed for cytotoxicity via the MTT assay and antiviral activity against DENV serotype 2 using flow cytometry and plaque formation assays. The 10–30 kDa peptide fraction, at 150 and 300 μg/mL concentrations, demonstrated no cytotoxicity and significantly reduced the percentage of infected cells and viral titers. These findings suggest that peptides derived from *Phaeodactylum tricornutum* exhibit promising antiviral activity against dengue virus serotype 2, potentially contributing to developing new therapeutic approaches for dengue.

## 1. Introduction

Dengue is a viral infection caused by the dengue virus (DENV) that represents a significant global health concern, with 400 million cases reported annually [1,2,3]. The virus is mainly transmitted through mosquito bites of *Aedes* species and comprises four serotypes (DENV 1 to 4) [4,5]. Symptoms of the disease can range in intensity from mild to severe, including high fever, severe headache, joint and muscle pain, and, in some cases, hemorrhagic fever or shock, which can be potentially fatal [1,6]. Early detection and medical attention are crucial for mitigating dengue fever complications. Research focuses on developing effective antiviral therapies, prioritizing symptom relief and supportive care. The lack of effective antiviral therapies underscores the urgent need for further development.

Antiviral peptides offer potential avenues for targeted treatments, providing insights into new therapeutic strategies. In this context, microalgae, photosynthetic microorganisms found in various aquatic environments, have recently gained significant attention as a source of bioactive compounds, particularly in developing new therapeutic agents. Previous research has reported the antiviral potential of microalgae derivatives, such as sulfated polysaccharides, carotenoids, polyunsaturated fatty acids, phenolic compounds, proteins, and peptides [7,8,9].

Among these derivatives, peptides hold the highest potential due to their unique mechanisms of action, high specificity, and lower likelihood of inducing resistance in viral pathogens. Peptides derived from microalgae can interfere with viral entry, replication, and assembly, making them promising candidates for antiviral drug development [10,11,12]. Their relatively simple synthesis and modification processes enhance their appeal as versatile and potent antiviral agents. Studies have shown that peptides from microalgae can effectively inhibit a range of viruses, including herpes simplex virus, HIV, and influenza virus, by disrupting the viral envelope or inhibiting viral enzymes critical for replication [10,11,12]. These antiviral peptides function through diverse mechanisms, such as binding to viral particles and preventing their attachment to host cells, blocking the fusion of viral membranes with host cell membranes, and interfering with the viral life cycle at various stages, including entry, replication, assembly, and release. For instance, specific peptides have been found to inhibit the activity of reverse transcriptase and protease enzymes in HIV, crucial for viral replication and maturation [13].

Moreover, the broad-spectrum antiviral activities of these peptides mean they can target multiple types of viruses, offering a versatile approach to combating viral infections. This versatility is particularly advantageous in the face of emerging viral pathogens and the frequent mutations in viruses like influenza, which can render traditional antiviral drugs less effective [14,15]. Also, microalgal peptides often exhibit low toxicity to host cells, which is critical for their potential therapeutic applications. This low toxicity is attributed to their high specificity for viral components, minimizing adverse effects on human cells [16,17].

Furthermore, the production of antiviral peptides from microalgae is sustainable and cost-effective. Microalgae can be cultured on a large scale using relatively simple and inexpensive methods, and they have a rapid growth rate, which facilitates the efficient production of bioactive compounds [18]. This scalability and advances in biotechnological techniques for peptide extraction and purification enhance the feasibility of developing microalgal peptides into pharmaceutical agents [19,20].

One microalga particularly rich in high-quality proteins is *Phaeodactylum tricornutum* (Bohlin, 1897). It can also proliferate rapidly and is relatively easy to cultivate, making it a viable option for harnessing its proteins [21]. Despite this, its antiviral potential has not yet been extensively studied. However, extracts derived from other microalgae, such as *Arthrospira maxima*, have shown antiviral effects against other flavivirus like Zika, suggesting that *P. tricornutum* might possess significant antiviral properties [12].

This paper presents the findings of a study on the separation and effectiveness of antiviral peptides derived from *P. tricornutum* against dengue virus serotype 2. The study aims to contribute to current efforts in addressing the lack of effective treatment for the dengue virus by exploring the potential of microalgae-derived peptides as a novel therapeutic approach.

## 2. Results

### 2.1. Cell Viability Assay of Phaeodactylum tricornutum Derived Peptides 

A comprehensive cell viability assay was conducted to evaluate the cytocompatibility of different molecular weights (<3 kDa, 3–5 kDa, 5–10 kDa, and 10–30 kDa) across a gradient of concentrations ranging from 0 to 1000 µg/mL. The assay was performed to ensure that the antiviral activity observed in subsequent experiments was not a consequence of cytotoxic effects. Using the mixed-effects model analysis, a significant main effect of concentration on cell viability (*p* < 0.0001), with no significant interaction between peptide size and concentration (*p* = 0.6710), was observed, indicating that peptide size did not modulate the effect of concentration on cell viability. Sidak’s multiple comparisons test substantiated the non-significant impact of concentration on cell viability within each peptide size category, emphatically validating the non-cytotoxic nature of the peptides at the concentrations tested.

Additionally, a random effects model corroborated the lack of significant cytotoxicity at the tested concentrations, which showed minimal variability attributable to the subject (SD = 0.05008), indicating that the intrinsic variability in cell viability was not a confounding factor.

**Figure 1:**
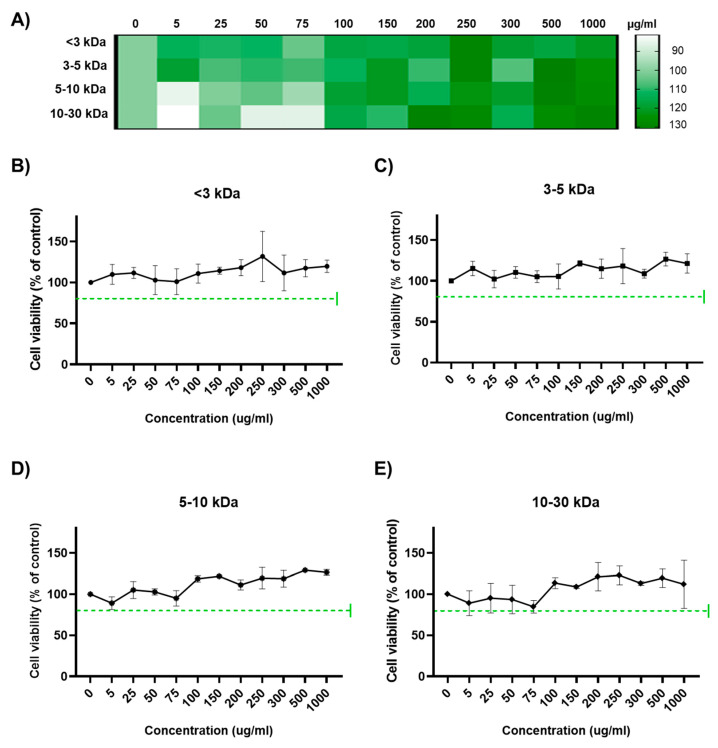
Impact of peptide concentration on cell viability across different molecular weights. Peptide-induced cytotoxicity was assessed across a concentration range of 0 to 1000 µg/mL for peptides of different molecular weights. (**A**) Heat map indicating the cytotoxic effects of protein hydrolysates according to their concentration. The colors indicate the relative levels of cytotoxicity; greater coloration corresponds to higher cell viability. The dotted line indicates 80% (to consider the safe concentrations to use). (**B**) <3 kDa, (**C**) 3–5 kDa, (**D**) 5–10 kDa, and (**E**) 10–30 kDa. Cell viability is expressed as a percentage of the untreated control, denoting the absence of significant cytotoxicity. Data are mean ± standard error of the mean (SEM) from three independent experiments.

Figure 1 shows the findings from the cell viability assay. The overall results are represented in the heatmap (panel A), arranged by peptide size and concentration from smallest to largest, where color intensity represents relative cytocompatibility; the higher the color intensity, the greater the cell viability, starting at 80% as all values exceed this threshold. Panel B, C, D, and E individually correspond to peptides with molecular weights of <3 kDa, 3–5 kDa, 5–10 kDa, and 10–30 kDa, respectively. The mean cell viability in all graphs did not significantly deviate from the untreated control, as illustrated by the error bars denoting the standard deviation based on triplicate measurements (Figure 1B–E). Additionally, the green dotted line indicates that cell viability remained well above 80% for all tested concentrations and molecular weights, suggesting a high degree of cytocompatibility.

### 2.2. Inhibition Assays by Flow Cytometry

**Figure 2:**
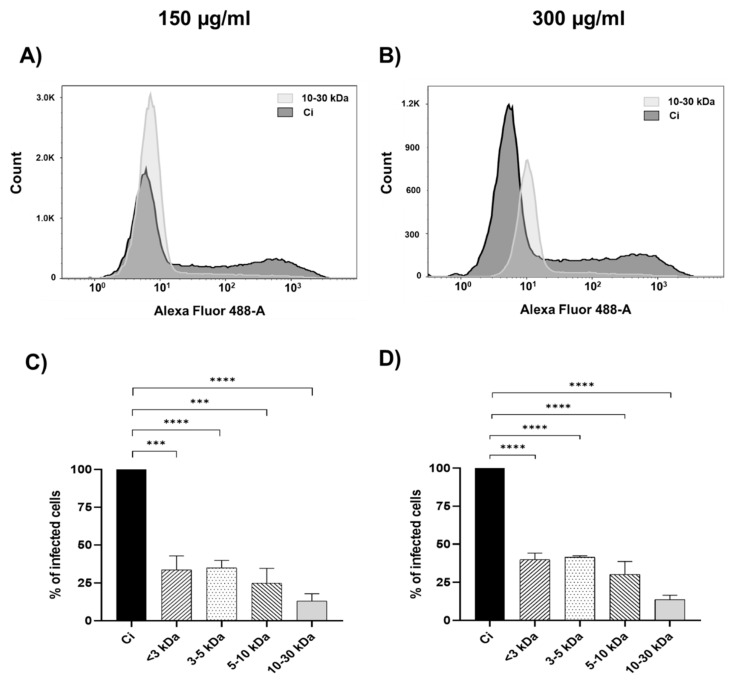
Efficacy of peptide fractions on dengue virus infection in Huh-7 cells assessed by flow cytometry. Panels show histograms comparing the infection control and high molecular weight peptides (10–30 kDa) at 150 (**A**) and 300 μg/mL (**B**) concentrations, respectively. Panels represent the percentage of infected cells at peptide concentrations of 150 μg/mL (**C**) and 300 μg/mL (**D**), respectively. Asterisks denote statistical significance: *** *p* < 0.001, **** *p* < 0.0001.

The antiviral activity of *P. tricornutum* peptide fractions against dengue virus serotype 2 (DENV-2) was assessed using flow cytometry. The Huh-7 cells were treated with peptides of various molecular weights (<3 kDa, 3–5 kDa, 5–10 kDa, and 10–30 kDa) at two concentrations (150 μg/mL and 300 μg/mL), and the percentage of infected cells was quantified. The results are presented in Figure 2 for peptide concentrations of 150 μg/mL and 300 μg/mL, respectively.

Figure 2A,B show a histogram comparing the infection control and the high molecular weight peptides (10–30 kDa) at 150 μg/mL and 300 μg/mL, respectively. At a concentration of 150 μg/mL (Figure 2C), treatment with peptides revealed a size-dependent reduction in the percentage of infected cells. The <3 kDa and 3–5 kDa fractions showed a modest decrease compared with the infection control, which displayed a near-total infection rate. In contrast, the 5–10 kDa and 10–30 kDa fractions demonstrated a more pronounced reduction in infected cells, indicating a correlation between peptide size and antiviral efficacy. This observation is supported by the histograms corresponding to the flow cytometry analysis (Figure 2A), which provide a fluorescence intensity distribution related to the number of infected cells.

The trend was maintained for the higher concentration of 300 μg/mL (Figure 2D), with the 10–30 kDa fraction showing the most significant reduction in infection rates. The accompanying histograms (Figure 2B) illustrate the shift towards lower infection prevalence across the samples treated with higher molecular weight peptides. The two-way ANOVA analysis indicated no significant interaction between peptide size and concentration (*p* = 0.9996), suggesting that the observed antiviral effects are predominantly attributable to the molecular size of the peptides rather than the concentration. Importantly, peptide size was a significant factor (*p* = 0.0026), while the concentration did not independently affect the outcomes (*p* = 0.7675). These statistical analyses confirm the size-dependent nature of the antiviral effect exhibited by the peptides.

The results consistently show that higher molecular weight peptides (10–30 kDa) at both concentrations tested (150 μg/mL and 300 μg/mL) achieve the most significant reduction in infection rates, underscoring the size-dependent efficacy of these antiviral peptides.

### 2.3. Viral Yield Reduction Efficacy at Varied Peptide Concentrations

The antiviral potential of peptide fractions against DENV-2 was systematically evaluated through plaque assays. These assays were designed to measure the viral yield and infer the inhibitory effects of peptides of varying molecular weights.

**Figure 3:**
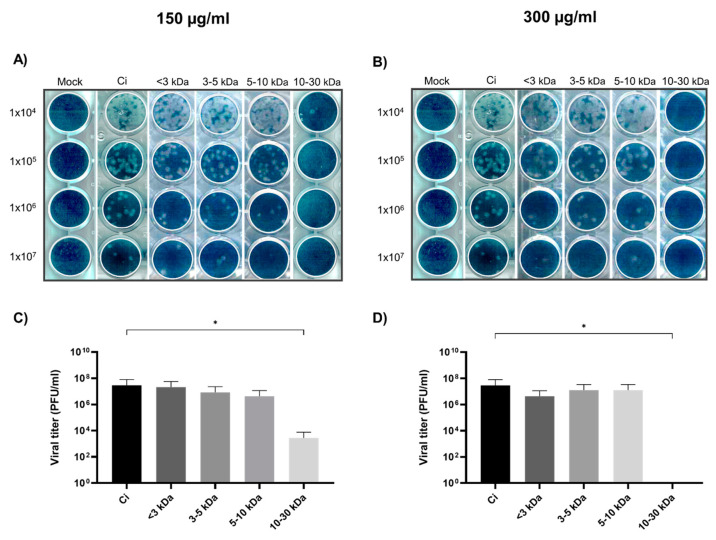
Antiviral efficacy of peptide fractions against dengue virus serotype 2 in plaque assays. Panels show the lytic plaques at four different dilutions for peptide concentrations of 150 μg/mL (**A**) and 300 μg/mL (**B**), respectively, with an evident reduction observed for the 10–30 kDa fraction at both concentrations and the viral titers at peptide concentrations of 150 μg/mL (**C**) and 300 μg/mL (**D**), respectively. Asterisks denote statistical significance: * *p* < 0.05.

Panels A and B show the lytic plaques at four different dilutions for peptide concentrations of 150 μg/mL and 300 μg/mL, respectively, with an evident reduction observed for the 10–30 kDa fraction at both concentrations. Plaque assays conducted at 150 μg/mL concentrations for the different peptide fractions exhibited a stratified inhibitory effect on viral replication. The highest molecular weight fraction (10–30 kDa) demonstrated the highest reduction in viral titer, suggesting peptide size-dependent inhibition. The viral yield reduction was notably substantial and statistically significant, particularly in the 10–30 kDa fraction, as depicted in Figure 3C.

Elevating the concentration to 300 μg/mL maintained the inhibitory trend, with no significant increase in antiviral effect, indicating a potential threshold beyond which additional concentrations do not further suppress viral yield. As illustrated in Figure 3D, the peptide fractions continued to demonstrate antiviral activity, with the >10–30 kDa fraction showing the most pronounced effect (Figure 3D).

Finally, a two-way ANOVA analysis was performed to discern the influence of peptide concentration on their antiviral efficacy. The study showed no significant interaction effect between the concentration and peptide size (*p* = 0.7730), indicating a consistent antiviral impact across the tested concentrations. This was particularly true for the peptide size, which proved to be a critical factor in the reduction of viral replication, with a highly significant effect (*p* = 0.0002). In contrast, alterations in concentration did not significantly influence the outcome (*p* = 0.9546), suggesting that the efficacy of the peptides was not dose-responsive within the tested range.

## 3. Discussion

This study aimed to assess the inhibitory effect of peptide fractions derived from *Phaeodactylum tricornutum* on DENV-2. An in vitro antiviral assay was conducted to determine the impact of the molecular weight on the antiviral activity of the peptides. The experiment used two different concentrations, 150 μg/mL and 300 μg/mL. Flow cytometry and plaque assays were used to assess the size-dependent activity, resulting in significant findings comprehensively. Our results demonstrated the possibility of antiviral properties in the peptides, especially for the higher molecular weights. In addition, the statistical analysis revealed that peptide size significantly decreased viral replication by two-way analysis of variance (ANOVA). However, the results did not differ with different concentrations. Concluding that the molecular weight range of peptides from microalga *P. tricornutum* could be essential to their efficacy against DENV-2.

The significant antiviral activity observed in higher molecular weight peptide fractions in our study aligns with previous studies, suggesting a correlation between molecular weight and antiviral potency. For example, Chew et al. [22] reported that larger peptide molecules tend to exhibit more substantial antiviral effects, potentially due to their enhanced ability to interfere with viral entry and replication processes. Similarly, Panya et al. [23] and Pujol et al. [24] documented the superior antiviral properties of larger peptides, emphasizing their efficacy in inhibiting a wide range of viruses, including dengue.

Panya et al. demonstrated that larger peptides from the medicinal plant *Acacia Catechu* exhibited significant antiviral activity against the dengue virus. These peptides are effectively bound to viral particles, preventing their attachment and entry into host cells [23]. This mechanism mirrors our findings with *P. tricornutum* peptides, where higher molecular weight fractions showed superior efficacy in reducing viral titers and infected cell percentages. For example, the 10–30 kDa fraction reduced viral titers by over 70% at 300 μg/mL, underscoring consistent results across different natural sources and highlighting the importance of peptide size in antiviral activity.

In another study, Pujol et al. investigated sulfated polysaccharides from algae and found potent inhibitory effects against dengue virus [24]. The structural complexity of these polysaccharides, akin to higher molecular weight peptides, was crucial for their antiviral function. This supports our findings, as the 10–30 kDa peptides from *P. tricornutum* consistently reduced the percentage of infected cells by approximately 65% at both tested concentrations (150 and 300 μg/mL). Moreover, Gao et al. provided a comprehensive overview of antiviral peptides with in vivo activity, highlighting their stability and minimal side effects [25]. Their work aligns with our findings that peptides from *P. tricornutum* exhibit low cytotoxicity, making them promising candidates for safe and effective antiviral therapies. In our cytotoxicity assays, these peptides showed no significant cytotoxic effects at concentrations up to 1000 μg/mL, reinforcing their potential for therapeutic use.

Furthermore, Lee et al. and Guntamadugu et al. used molecular docking and simulation studies to identify promising antiviral peptides based on their binding stability and interactions with viral proteins [26,27]. Their research supports the notion that peptides with higher binding affinity and stability, typically those with larger molecular weights, are more effective in inhibiting viral activity. For example, Lee et al. identified a peptide that demonstrated cell protection and post-infection inhibition across all four DENV serotypes, with direct virus-inactivating effects observed mainly against DENV-2, DENV-3, and DENV-4 [26]. This indicates a broad-spectrum antiviral potential, which is crucial for simultaneously tackling multiple serotypes of dengue virus. Similarly, Guntamadugu et al. highlighted peptides like Indolicidin and Human Neutrophil peptide-1, which showed protein-peptide solid interactions, suggesting their utility in treating dengue virus infections by stabilizing protein structures critical for viral replication [27]. These studies provide a mechanistic basis for our observation that higher molecular weight peptides from *P. tricornutum* exhibit more potent antiviral effects, as demonstrated by their significant reduction in viral replication.

In addition to peptide size, factors such as peptide sequence, secondary structure, and charge significantly influence antiviral efficacy. For instance, Recalde-Reyes et al. [28] and Hoffmann et al. [29] explored the specific mechanisms by which antiviral peptides inhibit viral entry and replication. Recalde-Reyes et al. identified peptides that could block protein-protein interactions necessary for viral invasion [28]. At the same time, Hoffmann et al. discussed the broad-spectrum antiviral activity of peptides that inhibit virus entry through interfacial activity [29]. These findings suggest that the efficacy of *P. tricornutum* peptides may also stem from their ability to interfere with critical viral processes, a hypothesis that warrants further investigation.

While this study demonstrates an apparent size-dependent antiviral effect, it is essential to consider additional factors that could influence the efficacy of peptides. Although the two-way ANOVA did not show a significant interaction between peptide size and concentration, the concentrations tested may not have varied enough to capture potential concentration-dependent effects. Moreover, this study did not evaluate the peptide sequence, secondary structure, and charge, which can impact antiviral activity [30].

Another essential aspect that needs to be addressed in this study is the specific mechanism by which the peptides exert their antiviral effects. Understanding the precise interactions between the peptides and the viral components is crucial for optimizing their therapeutic potential. The chemical composition of the protein hydrolysates extracted from *Phaeodactylum tricornutum* includes a variety of essential and non-essential amino acids, with a notable presence of water-soluble peptides. This is due to the protein extraction technique, which focuses on precipitating and recovering the water-soluble proteins, which were then subjected to enzymatic hydrolysis [31]. As stated by Sørensen et al., these water-soluble peptides predominantly contain amino acids with polar or charged properties, allowing favorable interactions with water molecules. The predominant amino acids include polar uncharged amino acids (serine and threonine), positively charged or essential amino acids (lysine, arginine, and histidine), and negatively charged or acidic amino acids (aspartic acid and glutamic acid) [31]. Combining these amino acids in a peptide sequence imparts water solubility, facilitating dissolution in aqueous solutions and enhancing their interaction with the aqueous environment [32].

Previously, have been observed that antiviral peptides can act against viruses through various mechanisms, which can be physical or chemical. These include binding to specific viral subunits, preventing viral entry into host cells, interacting with viral envelopes, and modulating the host’s immune pathways [33]. In this context, the findings showed that peptides with a molecular weight ranging from 10 to 30 kDa exhibit the most significant antiviral effect compared to other tested fractions. This molecular weight range suggests that the observed antiviral activity could be related to a mixture of peptides of different sizes, which might act synergistically. Larger peptides could interact with multiple binding sites or functional domains, and by working together, they enhance antiviral activity. Synergy implies that the peptides interact cooperatively, resulting in an antiviral effect more significant than the sum of the individual effects of each peptide. Previous studies have demonstrated that combinations of peptides yield better results than when tested alone [34].

Furthermore, 10 to 30 kDa peptides might interact with dengue virus proteins due to their size, allowing easier binding to specific sites on these proteins. The structural glycoproteins of the virus, such as prM (15 kDa) and E (53 kDa), have binding sites and critical regions that appropriately sized peptides can recognize and block. Additionally, non-structural proteins like NS3 (70 kDa) and NS5 (103 kDa) contain functional domains that can be targeted for peptides, interfering with their enzymatic activity essential for viral replication. These proteins have basic residues that can interact or bind selectively with negatively charged peptides, acting as competitive inhibitors and resulting in the inactivation of their catalytic function. This interference would affect the later stages of the viral cycle, reducing viral particle production. Previous studies have reported peptides and peptidomimetics that interact with the NS2B-NS3 protease of DENV2 [35,36]. Previous research indicates that antiviral peptides can function through various mechanisms, such as blocking viral entry, inhibiting viral replication, and preventing the assembly of new viral particles [26,27]. For example, antiviral peptides derived from the human CD44 receptor have been shown to inhibit protein-protein interactions critical for dengue virus entry and replication [28]. Therefore, it is reasonable to conclude that combining these factors suggests that a mixture of peptides with varied and more significant molecular weights may provide an effective strategy for inhibiting viral replication due to each peptide’s specific properties and synergistic interactions.

Dengue has long been a primary public health concern, with periodic outbreaks causing significant morbidity and mortality. The virus’s rapid spread, especially in tropical and subtropical regions, underscores the need for new antiviral strategies. Despite extensive efforts to control the mosquito vector *Aedes aegypti*, increasing resistance to chemical insecticides has rendered traditional methods less effective [37]. Consequently, there is an urgent need for novel antiviral agents, particularly those derived from natural sources like microalgae. Products from microalgae have shown efficacy against other viruses and are generally safer and less likely to generate resistance compared to synthetic compounds [12]. In this context, the present study’s findings are particularly promising. The higher molecular weight peptides from *P. tricornutum* demonstrated significant antiviral activity against DENV-2 and maintained this effect across the concentrations tested. This highlights the potential of these peptides as effective antiviral agents. Notably, peptides with higher molecular weights (10–30 kDa) exhibited the most significant reduction in viral replication, underscoring their therapeutic potential. 

A variety of bioactive compounds against different serotypes of the dengue virus have already been reported from natural sources, such as sulfated polysaccharides, flavonoids, alkaloids, terpenoids, polycyclic quinones, phenolic compounds, among others [24,38]. Additionally, numerous peptides targeting different structures of the dengue virus, directed at structural and non-structural proteins, have been reported from natural and synthetic origins [22]; however, this is the first report of peptides with antiviral effects (against DENV) derived from *P. tricornutum* microalgae.

Future research should focus on elucidating the mechanisms by which these peptides inhibit DENV-2, exploring factors such as peptide sequence, structural properties, and interactions with viral proteins. Investigating the effects of these peptides in vivo and in combination with other antiviral agents could provide valuable insights into their potential as part of a comprehensive antiviral strategy.

In conclusion, this study demonstrates that peptides derived from *P. tricornutum* exhibit significant antiviral activity against dengue virus serotype 2, with higher molecular weight peptides showing the best efficacy. These findings highlight the importance of peptide size in determining antiviral effectiveness and suggest that peptides with higher molecular weights have significant potential as therapeutic agents for treating DENV-2 infections and possibly other serotypes and viruses. This work represents a starting point, encouraging further studies to examine different factors, such as specific mechanisms of action and their effects in vivo models, to provide a more comprehensive understanding of their therapeutic potential.

## 4. Materials and Methods

### 4.1. Biologic Materials and Reagents

Microalgae *P. tricornutum* is part of the strain collection of the Molecular Physiology Laboratory at the Centro de Investigación Aplicada a la Salud Pública (CIASAP), Faculty of Medicine, Autonomous University of Sinaloa, Mexico. This strain was provided by the culture collection of the Centro de Investigación Científica y Educación Superior de Ensenada (CICESE) in Baja California, Mexico. The Huh-7 cell line from human hepatoma, BHK-21 cells, and DENV serotype 2 (DENV-2) virus were provided by the Department of Infectomics and Molecular Pathogenesis of CINVESTAV, Zacatenco, Mexico. Vitamins, alcalase, and other chemical reagents were obtained from Sigma-Aldrich (St. Louis, MO, USA).

### 4.2. Microalgae Culture Condition and Biomass Processing

*P. tricornutum* was cultured in an F/2 medium, as reported by Guillard and Ryther (1962) [39], under conditions previously described by Norzagaray-Valenzuela et al. (2017) [40], with minor modifications. Briefly, the microalga was cultured at 22 °C with 1% CO_2_ under continuous light (irradiance of 120–130 μmol photons m^−2^ s^−1^). The culture was harvested in the late log phase of growth using chitosan as a flocculant and then centrifuged at 4000 rpm for 5 min. Microalgal lipids were extracted following the modified Bligh and Dyer (1959) methodology [41]. Fifty grams of sample was exposed to 100 mL of ethyl acetate with ultrasonication (200 W power, 24 kHz frequency, 40% amplitude) on ice for one hour, using an ultrasonic homogenizer processor UP250S (Lawson Scientific, Hangzhou, Zhejiang, China). Subsequently, the mixture was stirred for 24 h at room temperature and was centrifuged at 4000 rpm for 2 min. Residual biomass of microalgae was collected, dried at 45 °C for 24 h, ground into powder, and stored at 4 °C.

### 4.3. Protein Concentration

Protein extraction was performed using the salting-out method described by Safi et al. (2014) [42] and Afify et al. (2018) [43]. Forty grams of residual biomass was dissolved in 500 mL of 2 M NaOH and stirred at 40 °C for 2 h. The mixture was centrifuged at 4000 rpm for 10 min at 20 °C, and the supernatant was collected while the precipitate was discarded. Proteins were precipitated from the supernatant by adjusting the pH to 3 using 5 N and 1 N HCl, followed by centrifugation at 4000 rpm for 20 min at 20 °C. The supernatant was discarded, and the precipitate was resuspended in 0.01 M NaOH and then neutralized with 1 N HCl to pH 7.

### 4.4. Protein Hydrolysis

Protein hydrolysis was conducted using the method of Humisky and Aluko (2007) [40], with modifications. The process was initiated by adding Alcalase^®^ at an enzyme-to-substrate (E/S) ratio of 4%, corresponding to 0.40 µL of enzyme per mg of protein, as quantified using the BCA Kit (Pierce^®^ Microplate BCA Protein Assay Kit–Reducing Agent Compatible, Central Jakarta, Indonesia). The reaction was maintained at optimal conditions for Alcalase^®^ activity: pH 9.0 and 50 °C for 60 min, utilizing a benchtop pH meter and a thermostatted hotplate stirrer (Mettler-Toledo, Columbus, OH, USA). Enzymatic activity was halted by adjusting the solution to pH 4.0 using 2 N HCl and incubating it in a water bath at 94 °C for 15 min. The mixture was centrifuged at 4000 rpm for 30 min, and the supernatant was collected and stored at −4 °C until further use.

### 4.5. Differential Fractionation of Protein Hydrolysates

The separation of protein hydrolysates into distinct molecular weight fractions was performed using ultrafiltration, following a modified version of the methodology by Hou et al. (2019) [44]. Various filtration devices with specific molecular weight cut-offs (MWCOs) were employed, including Amicon^®^ Ultra-15 centrifugal filters (Millipore, Billerica, MA, USA) with MWCOs of 30, 10, and 3 kDa, and Vivaspin^®^ Turbo centrifugal concentrators with a 5 kDa MWCO (Sartorius, Goettingen, Germany). Initially, 10 mL of the hydrolysate was processed through the 30 kDa filter, centrifuged at 3000× *g* at four °C for 20 min, and the >30 kDa retentate was recovered. The filtrate was sequentially passed through the 10 kDa, 5 kDa, and 3 kDa filters, with the 10–30 kDa, 5–10 kDa, and 3–5 kDa retentates being recovered. The final filtrate of <3 kDa was also recovered. All fractions were lyophilized and stored at −80 °C until use.

### 4.6. Cell Culture, Virus, and Reagents

Huh-7 and BHK-21 cells were cultured in Advanced DMEM supplemented with 2 mM glutamine, 50 μg/mL streptomycin, 50 IU/mL penicillin, 8% fetal bovine serum (FBS), and 1 mL/L amphotericin B at 37 °C in a 5% CO_2_ atmosphere. DENV serotype 2 (New Guinea strain) propagation was conducted using CD-1 suckling mice brains provided by Unidad de Producción y Experimentación de Animales de Laboratorio (UPEAL-Cinvestav) Cinvestav. 

### 4.7. Cell Viability Assay

Cell viability assays were conducted to evaluate the cytocompatibility of peptide fractions. Huh-7 cells were treated with increasing concentrations of peptide fractions (0 to 1000 µg/mL). The MTT method (3-(4,5-dimethylthiazol-2-yl)-2,5-diphenyltetrazolium bromide) was used according to the manufacturer’s instructions. The assay was evaluated by spectrophotometry (BioTek ELx800TM, Winooski, VT, USA), measuring absorbance at 540 nm, interpreted as the proportion of viable cells.

### 4.8. Antiviral Activity Assays by Flow Cytometry

The antiviral activity of the peptides against DENV-2 was quantified via flow cytometry. For the assessment of the percentage of DENV-2 infection, Huh-7 cells were seeded in 12-well plates and subjected to treatment with various peptide fractions (<3 kDa, 3–5 kDa, 5–10 kDa, and 10–30 kDa). The cells were treated with these different molecular weight peptide fractions and were analyzed for the percentage of DENV-positive cells. Post-treatment, cells were harvested and fixed using 1% formaldehyde to preserve cellular integrity and permeabilized with a solution containing 0.1% saponin, 1% FBS, and 1x PBS for 20 min to allow for antibody penetration.

Specific primary antibodies were employed to target viral protein anti-prM-E (2H2), which were incubated with the cells for two hours at room temperature. Following primary antibody incubation, cells were washed and then incubated with fluorescently labeled secondary antibodies (goat anti-mouse Alexa Fluor 488, Life Technologies, Carlsbad, CA, USA). Flow cytometry was utilized to quantify the percentage of infected cells by analyzing fluorescence intensities to determine the efficacy of peptide treatments in reducing viral infection rates. This analysis was performed using a BD LSR Fortessa™ flow cytometer (Franklin Lakes, NJ, USA), with subsequent data analysis conducted using FlowJo version 10 software. 

### 4.9. Viral Yield Reduction Assays

Viral yield was assessed using plaque assays on BHK-21 cells monolayers (2.5 × 10^5^ cells at 80% confluence in 24-well plates), which were infected with serially diluted supernatant from the infection and peptide-treatment assay, and then incubated for 2 h at 37 °C. Overlay medium (0.5 mL; MEM supplemented with 7.5% FBS, 1% carboxymethylcellulose, and antibiotics) was added, and the plates were incubated for five days at 37 °C in a 5% CO_2_ humidified atmosphere. Finally, the medium was removed, and the monolayers were stained with naphthol blue-black (0.1% naphthol blue-black, 0.165 M sodium acetate, and 6% acetic acid) for 2 h at room temperature. The viral yield from three independent experiments was measured and expressed in the log of plaque-forming units (PFU)/mL.

### 4.10. Statistical Analysis

All statistical analyses were carried out using GraphPad Prism version 9.0. Before conducting parametric tests, data normality and variance homogeneity were verified using the Shapiro-Wilk and Levene’s tests, respectively. Where assumptions of normality and equal variances were met, differences between multiple groups were analyzed using one-way ANOVA, supplemented with Tukey’s post hoc test for pairwise comparisons. For data not meeting these assumptions, non-parametric equivalents were employed, specifically the Kruskal-Wallis test, followed by Dunn’s post hoc test. In cases involving multiple independent variables, two-way ANOVA was utilized to discern the effects of interactions between factors such as peptide size and concentration on the measured outcomes. Statistical significance was considered as *p*-value ≤ 0.05. The results were expressed as mean ± standard deviation. For flow cytometry data, where multiple measurements were taken from the same sample, a mixed-effects model was used to account for intra-sample correlation, with Sidak’s multiple comparisons test adjusting for multiple comparisons.

## 5. Conclusions

In conclusion, this study demonstrates that peptides derived from *Phaeodactylum tricornutum* exhibit significant antiviral activity against dengue virus serotype 2, with higher molecular weight peptides showing the most considerable efficacy. These findings highlight the importance of peptide size in antiviral effectiveness and suggest that peptides with higher molecular weights have significant potential as therapeutic agents for treating DENV-2 infections and possibly other serotypes and viruses. This work represents a starting point, encouraging further studies to examine other factors, such as specific mechanisms of action and their effects in vivo models, to provide a more comprehensive understanding of their therapeutic potential.

## Data Availability

The datasets generated and analyzed during the study are available from the corresponding authors upon reasonable request.
